# Sex Differences in the Association of Obesity With Prediabetes and Dyslipidemia Among Adolescents: A Cross‐Sectional Study

**DOI:** 10.1002/osp4.70096

**Published:** 2025-11-11

**Authors:** Ali H. Ziyab, Aishah Saadallah, Zainab Almousa, Mohammad Almari, Thamer Alessa

**Affiliations:** ^1^ Department of Community Medicine and Behavioral Sciences College of Medicine Kuwait University Safat Kuwait; ^2^ Department of Health Policy and Management College of Public Health Kuwait University Safat Kuwait; ^3^ Division of Endocrinology, Diabetes & Metabolism Jaber Al‐Ahmad Hospital Ministry of Health Sulaibikhat Kuwait; ^4^ Dasman Diabetes Institute Dasman Kuwait

**Keywords:** adolescents, children, dyslipidemia, obesity, prediabetes, sex

## Abstract

**Background:**

Limited knowledge exists on whether obesity during early life stages demonstrates sex‐specific associations with cardiometabolic conditions. Therefore, this study aimed to determine if the association of obesity with prediabetes and dyslipidemia differs according to sex among adolescents.

**Methods:**

Adolescents aged 14–19 years were enrolled in a cross‐sectional study. Capillary blood was used to measure glycated hemoglobin and lipids. Prediabetes and dyslipidemia were determined according to international guidelines. Associations and statistical interactions (body mass index‐for‐age × sex) were evaluated using multivariable logistic regression models.

**Results:**

Data from a total of 1584 adolescents (826 female participants) were analyzed in the current report. Obesity (38.6% vs. 24.4%) and dyslipidemia (54.2% vs. 36.7%) were more prevalent in male than female participants; however, prediabetes prevalence did not differ between male and female participants (34.8% vs. 33.8%). The association between obesity and prediabetes differed according to sex (*P*_interaction_ = 0.046), with obesity showing a stronger association among female participants (adjusted odds ratios [aOR]: 3.24; 95% confidence interval [CI]: 2.25, 4.66) compared with male participants (aOR: 1.64; 95% CI: 1.12, 2.39). However, obesity showed a stronger association with dyslipidemia among male participants (aOR: 2.74; 95% CI: 1.93, 3.90) compared with female participants (aOR: 1.49; 95% CI: 1.04, 2.13; *P*_interaction_ = 0.016).

**Conclusion:**

Obesity demonstrated sex‐specific associations with cardiometabolic conditions in adolescents, showing a stronger association with prediabetes in females but with dyslipidemia in males.

## 1 Introduction

Global prevalence estimates in 2021 reported that 18.1% of children aged 5–14 years and 20.3% of adolescents aged 15–24 years were affected by overweight and obesity [1]. Obesity is associated with all‐cause and cause‐specific mortality as well as a range of chronic conditions, including diabetes and dyslipidemia [2, 3, 4]. More alarming is the increasing trends in childhood obesity, which predispose children and adolescents to early‐life cardiometabolic complications [5, 6]. Differences between the sexes in how diseases develop, present clinically, progress, and respond to medications are receiving growing attention in scientific research [7]. Specifically, accumulating evidence suggests the existence of sex differences in cardiometabolic disease risk [8, 9, 10]. For instance, type 2 diabetes mellitus (T2DM) is more frequently diagnosed in men compared with age‐matched premenopausal women, with this sex‐related difference in T2DM risk narrowing between men and age‐matched postmenopausal women [11, 12].

Among different factors that may explain the observed sex differences in cardiometabolic disease risk is body composition, which shows differential body fat distribution and mass in men and women [8, 13]. Men tend to accrue more visceral adipose tissue (VAT), which is more metabolically deleterious than subcutaneous adipose tissue (SAT), which is more common among premenopausal women [14, 15]. Hence, obesity may confer a sex‐specific differential risk when it comes to cardiometabolic diseases. Although sex differences in adipose tissue distribution exist early in life [8, 16], there is limited knowledge on whether obesity exerts sex‐specific effects on metabolic conditions at early life stages. A previous study investigated sex differences in prediabetes and insulin resistance markers among 1356 children and adolescents (2–19 years) with obesity who are at risk of diabetes. The findings revealed that insulin resistance was greater in girls with obesity than in boys with obesity, a female predominance that emerged even in pre‐puberty [17]. Another study reported sex differences in the association between the timing of obesity and incident T2DM: girls who developed obesity in early adolescence (< 16 years) compared to later (≥ 18 years) had a more than twofold increased risk for T2DM, while the same association was not found in boys [18]. Therefore, to address the existing gap in knowledge, this study tested the hypothesis that biological sex modulates the association between overweight and obesity and prediabetes and dyslipidemia among adolescents.

## 2 Methods

### 2.1 Study Design, Setting, and Participants

This cross‐sectional study was conducted in public high schools throughout the State of Kuwait, specifically enrolling students in grades 10–12 (age range, 14–19 years). Schools and students were selected using a cluster random sampling method that is previously described in details by Almari et al. [19]. A total of 1959 students participated in the study. A total of 375 subjects were excluded from the current analysis: 97 with a prior diabetes diagnosis, 17 subjects with undiagnosed diabetes (no prior diagnosis but HbA1c [glycated hemoglobin] of ≥ 6.5%), and 261 subjects who did not have lipid results. After exclusions, data from a total of 1584 students were analyzed in the current study. The study was approved by the Ethics Committee at Kuwait University Health Sciences Center (Approval No.: VDR/EC/3067). Parents or legal guardians provided written informed consent before enrolling adolescents in the study. This study uses the method of Almari et al. [19, 20] and Ziyab et al. [21], with the method description partly reproducing their wording.

### 2.2 Anthropometric Measurements

Standardized procedures were used to measure height (cm) and weight (kg) [19]. As body mass index (BMI) changes with age during growth, BMI‐for‐age *z*‐scores were calculated using the World Health Organization growth reference for children and adolescents aged 5–19 years [22]. BMI‐for‐age *z*‐scores were classified as: underweight (< −2 SD), normal weight (−2 to 1 SD), overweight (> 1 to 2 SD), and obesity (> 2 SD) [22].

### 2.3 Assessment of Prediabetes and Dyslipidemia

Capillary blood samples were analyzed without fasting to assess HbA1c and lipid parameters using the Cobas b 101 point‐of‐care device (Roche Diagnostics, Mannheim, Germany). Two discs were used: one disc for HbA1c and a second disc for lipids. The lipid disc provides measurements for total cholesterol (TC), high‐density lipoprotein cholesterol (HDL‐C), and triglyceride (TG) levels, while also providing a calculated low‐density lipoprotein cholesterol (LDL‐C) value using the Friedewald formula given that TG < 4.52 mmol/L [23]. Non–HDL‐C cholesterol was calculated as TC minus HDL‐C. Based on the manufacturer's evaluation [22, 24], the Cobas b 101 device passed the National Glycohemoglobin Standardization Program accuracy grading criteria for HbA1c [25] and lipid testing conformed to the National Cholesterol Education Program guidelines [26].

Prediabetes was defined using the American Diabetes Association criteria as HbA1c 5.7%–6.4% (39–47 mmol/mol) [27]. Lipid values were assigned to one of three categories, including acceptable (normal), borderline, or abnormal, as determined from pediatric standard reference guidelines [28, 29]. Normal, borderline (high or low), and abnormal values were established using the following cut‐offs (in mmol/L), respectively: TC: < 4.3, 4.3–5.1, > 5.1; triglycerides: < 1.0, 1.0–1.5, > 1.5; LDL‐C: < 2.8, 2.8–3.3, > 3.3; non‐HDL‐C: < 3.1, 3.1–3.7, > 3.7; HDL‐C: > 1.2, 1.0–1.2, < 1.0. Dyslipidemia was identified by the presence of at least one abnormal lipid value.

### 2.4 Covariates

Parents (or legal guardians) completed a structured questionnaire to report on the child's background and early‐life factors. The questionnaire assessed the child's nationality, their feeding history as it relates to breastfeeding (ever vs. never), mode of delivery as newborns, and birth order (among siblings). Parents provided information on smoking exposure in the home for the child and parental diabetes history. A parental history of diabetes was considered positive if either the mother or father reported having diabetes at any time. Household smoking exposure was defined positively by an affirmative answer to the question of whether anyone smokes cigarettes or a water‐pipe inside the home. For their own behaviors, students reported whether they had smoked at least one cigarette within a 30‐day interval, and how many days a week they engaged in moderately intense physical activities to cause them to breathe hard, as described by Ziyab et al. [19].

### 2.5 Statistical Analysis

SAS version 9.4 (SAS Institute, Cary, NC, USA) was used to perform the statistical analyses. A *p*‐value < 0.05 was used to indicate statistical significance. Descriptive analyses were performed by calculating frequencies and percentages of categorical variables. Since lipid levels were not normally distributed, continuous variables were summarized by calculating medians and interquartile ranges (25th and 75th percentiles). Univariable associations between categorical variables were evaluated by using chi‐squared test ( *χ* ^2^). Moreover, univariable associations between categorical and continuous variables were assessed by using the Wilcoxon rank‐sum test.

Associations between the BMI‐for‐age variable (exposure variable) and prediabetes, dyslipidemia, and individual lipid levels (outcome variables) were assessed in the total study sample and stratified by sex. Binary logistic regression models were evaluated to assess associations between the BMI‐for‐age variable and binary (two‐category) outcome variables (i.e., prediabetes and dyslipidemia status). Given that lipid variables have three categories (acceptable, borderline high/low, and abnormal), multinomial logistic regression models were used to evaluate associations between the BMI‐for‐age variable and lipid variables (acceptable category was set as the common reference). Moreover, ordinal logistic regression models were evaluated to assess associations between the BMI‐for‐age variable and the ordinal lipid outcomes. Hence, assuming that the odds of the outcome shift consistently from one category to the next category (e.g., moving from acceptable to borderline is equivalent to moving from borderline to abnormal). This is referred to as the proportionality assumption (also known as parallel lines assumption). The Brant test was used to assess whether the BMI‐for‐age variable (main exposure of interest) violates the proportional odds assumption when tested with each of the lipid variables. Moreover, the global “Score test” was evaluated, which tested the hypothesis that the model constrained by the proportional odds assumption is not significantly different from the model that does not constrain the odds parameters by the proportionality assumption. For all fitted models, the Hosmer‐Lemeshow goodness‐of‐fit test was evaluated.

Statistical interactions on a multiplicative scale were tested to determine whether the association between the BMI‐for‐age variable and the respective outcome variable differs according to sex by including a product term in the regression models (BMI‐for‐age × sex). The effect of variables showing potential univariable association (*p*‐value < 0.2) with BMI‐for‐age and/or sex was controlled for in all regression models. Adjusted odds ratios (aOR) and their 95% confidence intervals (CI) were estimated.

## 3 Results

The frequency of sociodemographic and lifestyle factors of the analyzed sample of high school students in Kuwait (*n* = 1584) is shown in Table 1, stratified by sex. The median age of the study participants was 16.0 years (IQR: 15.0, 17.0 years), with 37.0% of the participants aged ≤ 15 years old. Male compared to female participants were more likely to engage in physical activity, with 12.7% of male subjects compared to 4.7% of female subjects reporting engaging in physical activity five or more times per week (*p* < 0.001). Obesity was more prevalent in male than female participants (38.6% vs. 24.4%, *p* < 0.001). Age, nationality, breastfeeding history, mode of delivery, birth order, and household ETS exposure did not differ significantly between male and female subjects (Table 1).

**TABLE 1:** Sociodemographic characteristics and lifestyle factors of the enrolled high school students in Kuwait in the total analytical sample (*n* = 1584) and according to sex.

Variable	Total sample (*n* = 1584), % (*n*)	Males (*n* = 758), % (*n*)	Females (*n* = 826), % (*n*)	*p*‐value^a^
Sex				
Male	47.8 (758)	—	—	
Female	52.2 (826)	—	—	
Age (years)				
≤ 15	37.0 (582)	35.5 (269)	38.2 (313)	0.452
16−	29.5 (465)	29.6 (224)	29.5 (241)	
≥ 17	33.5 (528)	34.9 (264)	32.3 (264)	
Missing (*n*)	(9)	(1)	(8)	
Nationality				
Kuwaiti	94.4 (1493)	93.8 (710)	94.9 (783)	0.335
Non‐Kuwaiti	5.6 (89)	6.2 (47)	5.1 (42)	
Missing (*n*)	(2)	(1)	(1)	
Ever breastfed				
Yes	82.8 (1290)	82.7 (612)	83.0 (678)	0.882
Missing (*n*)	(27)	(18)	(9)	
Mode of delivery				
Vaginal	85.3 (1323)	84.1 (619)	86.4 (704)	0.206
Cesarean section	14.7 (228)	15.9 (117)	13.6 (111)	
Missing (*n*)	(33)	(22)	(11)	
Birth order				
First	24.2 (380)	25.0 (187)	23.5 (193)	0.896
Second	19.4 (304)	19.5 (146)	19.2 (158)	
Third	18.6 (293)	18.5 (139)	18.8 (154)	
Fourth or more	37.8 (593)	37.0 (277)	38.5 (316)	
Missing (*n*)	(14)	(9)	(5)	
Smoking status				
Never smoked	85.4 (1345)	70.4 (531)	99.3 (814)	< 0.001
Ever smoked	14.6 (229)	29.6 (223)	0.7 (6)	
Missing (*n*)	(10)	(4)	(6)	
Household ETS exposure				
Yes	50.5 (791)	50.4 (377)	50.7 (414)	0.914
Missing (*n*)	(19)	(10)	(9)	
Physical activity per week				
Never or occasionally	31.7 (500)	25.7 (194)	37.1 (306)	< 0.001
Once or twice	47.6 (751)	46.6 (351)	48.6 (400)	
Three or four times	12.2 (192)	15.0 (113)	9.6 (79)	
Five or more times	8.5 (135)	12.7 (96)	4.7 (39)	
Missing (*n*)	(6)	(4)	(2)	
BMI‐for‐age categories				
Underweight (< −2 SD)	2.4 (38)	2.8 (21)	2.1 (17)	< 0.001
Normal (−2 to 1 SD)	43.9 (695)	36.8 (279)	50.4 (416)	
Overweight (> 1 to 2 SD)	22.5 (356)	21.8 (165)	23.1 (191)	
Obesity (> 2 SD)	31.2 (494)	38.6 (293)	24.4 (201)	
Missing (*n*)	(1)		(1)	

Abbreviations: BMI, body mass index; ETS, environmental tobacco smoke; SD, standard deviation.

^a^ Calculated using chi‐squared test to compare frequencies in males and females.

Sex‐stratified frequencies of sociodemographic characteristics and lifestyle factors of the study participants across BMI‐for‐age categories are shown in Table 2. Among male participants, age, being ever breastfed, physical activity levels, and parental history of diabetes were associated with BMI‐for‐age categories (*p* < 0.05). For instance, the prevalence of obesity was higher among male participants who never/occasionally engaged in physical activity compared to participants who reported being physically active five or more times per week (47.4% vs. 21.9%, *p* < 0.001). Among female participants, the frequency of nationality and parental history of diabetes statistically significantly differed across BMI‐for‐age categories (*p* < 0.05). Female subjects who reported a parental history of diabetes were more likely to have obesity than those who did not report a parental history of diabetes (31.0% vs. 19.7%, *p* = 0.001; Table 2).

**TABLE 2:** Frequency of sociodemographic characteristics and lifestyle factors across body mass index (BMI)‐for‐age categories stratified by sex.

Variable	Males				Females			
	BMI‐for‐age categories, % (*n*)			*p*‐value^a^	BMI‐for‐age categories, % (*n*)			*p*‐value^a^
	Under/normal weight (≤ 1 SD)	Overweight (> 1 to 2 SD)	Obesity (> 2 SD)		Under/normal weight (≤ 1 SD)	Overweight (> 1 to 2 SD)	Obesity (> 2 SD)	
Age (completed years)								
≤ 15 (*n* = 269^c^, 313^d^)	36.1 (97)	25.3 (68)	38.6 (104)	0.006	53.1 (166)	22.0 (69)	24.9 (78)	0.760
16− (*n* = 224^c^, 241^d^)	34.4 (77)	19.6 (44)	46.0 (103)		53.1 (128)	25.3 (61)	21.6 (52)	
≥ 17 (*n* = 264^c^, 263^d^)	47.3 (125)	20.1 (53)	32.6 (86)		52.1 (137)	22.0 (58)	25.9 (68)	
Nationality								
Kuwaiti (*n* = 710^c^, 782^d^)	38.6 (274)	21.8 (155)	39.6 (281)	0.064	53.6 (419)	23.1 (181)	23.3 (182)	0.009
Non‐Kuwaiti (*n* = 47^c^, 42^d^)	55.3 (26)	19.2 (9)	25.5 (12)		33.3 (14)	23.8 (10)	42.9 (18)	
Ever breastfed								
Yes (*n* = 612^c^, 677^d^)	40.5 (248)	19.9 (122)	39.6 (242)	0.019	53.9 (365)	21.9 (148)	24.2 (164)	0.119
No (*n* = 128^c^, 139^d^)	34.4 (44)	31.2 (40)	34.4 (44)		46.0 (64)	29.5 (41)	24.5 (34)	
Mode of delivery								
Vaginal (*n* = 619^c^, 704^d^)	39.9 (247)	22.0 (136)	38.1 (236)	0.732	52.8 (372)	23.2 (163)	24.0 (169)	0.712
Cesarean section (*n* = 117^c^, 110^d^)	36.7 (43)	21.4 (25)	41.9 (49)		49.1 (54)	23.6 (26)	27.3 (30)	
Birth order								
First (*n* = 187^c^, 193^d^)	43.9 (82)	19.2 (36)	36.9 (69)	0.352	53.4 (103)	23.8 (46)	22.8 (44)	0.252
Second (*n* = 146^c^, 158^d^)	31.5 (46)	25.3 (37)	43.2 (63)		57.0 (90)	24.0 (38)	19.0 (30)	
Third (*n* = 139^c^, 154^d^)	36.7 (51)	22.3 (31)	41.0 (57)		55.8 (86)	22.1 (34)	22.1 (34)	
Fourth or more (*n* = 277^c^, 315^d^)	41.5 (115)	22.0 (61)	36.5 (101)		47.9 (151)	22.9 (72)	29.2 (92)	
Smoking status								
Never smoked (*n* = 531^c^, 813^d^)	37.3 (198)	22.6 (120)	40.1 (213)	0.122	52.6 (428)	23.0 (187)	24.4 (198)	0.261^b^
Ever smoked (*n* = 223^c^, 6^d^)	45.3 (101)	19.7 (44)	35.0 (78)		33.3 (2)	50.0 (3)	16.7 (1)	
Household ETS exposure								
Yes (*n* = 377^c^, 413^d^)	40.1 (151)	19.9 (75)	40.0 (151)	0.347	50.9 (210)	23.0 (95)	26.1 (108)	0.442
No (*n* = 371^c^, 403^d^)	38.5 (143)	24.3 (90)	37.2 (138)		53.9 (217)	23.8 (96)	22.3 (90)	
Physical activity per week								
Never or occasionally (*n* = 194^c^, 305^d^)	33.5 (65)	19.1 (37)	47.4 (92)	< 0.001	53.1 (162)	22.3 (68)	24.6 (75)	0.750
Once or twice (*n* = 351^c^, 400^d^)	37.0 (130)	23.7 (83)	39.3 (138)		52.5 (210)	24.3 (97)	23.2 (93)	
Three or four times (*n* = 113^c^, 79^d^)	40.7 (46)	23.0 (26)	36.3 (41)		50.6 (40)	19.0 (15)	30.4 (24)	
Five or more times (*n* = 96^c^, 39^d^)	58.3 (56)	19.8 (19)	21.9 (21)		53.8 (21)	28.2 (11)	18.0 (7)	
Parental diabetes								
Yes (*n* = 280^c^, 323^d^)	32.5 (91)	20.0 (56)	47.5 (133)	< 0.001	47.3 (153)	21.7 (70)	31.0 (100)	0.001
No (*n* = 459^c^, 492^d^)	43.8 (201)	23.3 (107)	32.9 (151)		56.1 (276)	24.2 (119)	19.7 (97)	

Abbreviations: BMI, body mass index; ETS, environmental tobacco smoke; SD, standard deviation.

^a^ Calculated using the chi‐squared test to compare frequencies across BMI‐for‐age categories.

^b^ Calculated using the Fisher's exact test.

^c^ Total male participants.

^d^ Total female participants.

Prevalence estimates and levels of prediabetes, dyslipidemia, and individual lipid measures in the total analytical sample and by sex are shown in Table 3. In the total analytical sample, prediabetes prevalence was estimated to be 34.3% (95% CI: 31.9, 36.6%), with no sex difference (*p* = 0.660). The proportion of participants affected by dyslipidemia (i.e., at least one abnormal level of TC, LDL‐C, non‐HDL‐C, HDL‐C, or triglycerides) in the total study sample was estimated to be 45.1% (95% CI: 42.6, 47.5%). A considerable proportion of the enrolled adolescents had abnormal levels of HDL‐C (19.7%) and triglycerides (33.0%; Table 3). The prevalence of dyslipidemia was statistically significantly higher among male participants compared with female participants (54.2% vs. 36.7%, *p* < 0.001). There were statistically significant differences in TC, HDL‐C, and triglyceride levels between male and female participants. For instance, more female than male participants had abnormal levels of TC (4.1% vs. 2.4%, *p* = 0.006). Male participants compared to female participants had higher prevalence of abnormal HDL‐C (31.8% vs. 8.6%, *p* < 0.001) and triglycerides (36.9% vs. 29.5%, *p* < 0.001; Table 3). Levels of LDL‐C and non‐HDL‐C did not vary between male and female participants.

**TABLE 3:** Prevalence of prediabetes and abnormal lipid levels and levels of glycated hemoglobin A1c (HbA1c) and lipids in the total analytical sample according to sex.

	Total (*n* = 1584),% (*n*)	Males (*n* = 758), % (*n*)	Females (*n* = 826), % (*n*)	*p*‐value
Prediabetes				
Yes (HbA1c: 5.7%–6.4%)	34.3 (543)	34.8 (264)	33.8 (279)	0.660^a^
HbA1c %, median (IQR)	5.50 (5.30, 5.70)	5.50 (5.30, 5.70)	5.50 (5.30, 5.70)	0.254^b^
Dyslipidemia				
Yes	45.1 (710)	54.2 (409)	36.7 (301)	< 0.001^a^
Missing (*n*)	(9)	(4)	(5)	
Total cholesterol (mmol/L)				
Acceptable (< 4.3)	84.3 (1335)	87.4 (662)	81.5 (673)	0.006^a^
Borderline (4.3–5.1)	12.3 (195)	10.0 (76)	14.4 (119)	
Abnormal (> 5.1)	3.4 (54)	2.6 (20)	4.1 (34)	
Median (IQR)	3.58 (3.19, 4.07)	3.42 (3.07, 3.92)	3.67 (3.30, 4.15)	< 0.001^b^
LDL‐C (mmol/L)				
Acceptable (< 2.8)	94.1 (1469)	93.3 (695)	94.7 (774)	0.415^a^
Borderline (2.8–3.3)	3.8 (60)	4.2 (31)	3.6 (29)	
Abnormal (> 3.3)	2.1 (33)	2.5 (19)	1.7 (14)	
Median (IQR)	1.72 (1.34, 2.12)	1.70 (1.32, 2.11)	1.74 (1.35, 2.14)	0.270^b^
Missing (*n*)	(22)	(13)	(9)	
Non‐HDL‐C (mmol/L)				
Acceptable (< 3.1)	87.2 (1380)	86.1 (653)	88.1 (727)	0.305^a^
Borderline (3.1–3.7)	9.5 (151)	9.9 (75)	9.2 (76)	
Abnormal (> 3.7)	3.3 (52)	4.0 (30)	2.7 (22)	
Median (IQR)	2.30 (1.95, 2.77)	2.30 (1.87, 2.79)	2.30 (2.01, 2.76)	0.192^b^
Missing (*n*)	(1)		(1)	
HDL‐C (mmol/L)				
Acceptable (> 1.2)	50.2 (795)	33.4 (253)	65.7 (542)	< 0.001^a^
Borderline (1.0–1.2)	30.1 (476)	34.8 (264)	25.7 (212)	
Abnormal (< 1.0)	19.7 (312)	31.8 (241)	8.6 (71)	
Median (IQR)	1.21 (1.03, 1.43)	1.10 (0.95, 1.27)	1.32 (1.14, 1.56)	< 0.001^b^
Missing (*n*)	(1)		(1)	
Triglycerides (mmol/L)				
Acceptable (< 1.0)	32.2 (508)	29.7 (224)	34.5 (284)	0.007^a^
Borderline (1.0–1.5)	34.8 (548)	33.4 (252)	36.0 (296)	
Abnormal (> 1.5)	33.0 (521)	36.9 (278)	29.5 (243)	
Median (IQR)	1.23 (0.91, 1.70)	1.28 (0.94, 1.79)	1.18 (0.89, 1.63)	0.012^b^
Missing (*n*)	(7)	(4)	(3)	

Abbreviations: HDL‐C, high‐density lipoprotein cholesterol; IQR, interquartile range; LDL‐C, low‐density lipoprotein cholesterol.

^a^ Calculated using chi‐squared test to compare frequencies in males and females.

^b^ Calculated using the Wilcoxon rank‐sum test to compare medinas in males and females.

Results of multivariable binary, multinomial, and ordinal logistic regression models testing the associations of the BMI‐for‐age variable with prediabetes and dyslipidemia measures are shown in Table 4. For all tested regression models, there was not enough evidence to indicate lack of fit (*p* > 0.05). Moreover, in all tested ordinal logistic regression models, the BMI‐for‐age variable did not violate the proportional odd assumption (Brant test: *p* > 0.05). Similarly, the global “Score test” was statistically nonsignificant for the tested ordinal logistic regression models (*p* > 0.05), indicating that the model constrained by the proportional odds assumption is not significantly different from the model that does not constrain the odds parameters by the proportionality assumption.

**TABLE 4:** Associations of body mass index (BMI)‐for‐age categories with prediabetes, dyslipidemia, and lipid status in the total study sample and stratified by sex.

Outcome	Total sample		Males		Females		*P*_Interaction_ ^b^
	Overweight^a^	Obesity^a^	Overweight^a^	Obesity^a^	Overweight^a^	Obesity^a^	
Prediabetes							
aOR^c^_[Yes vs. No]_, (95% CI)	1.29 (0.97, 1.72)	2.53 (1.95, 3.27)^f^	0.89 (0.57, 1.39)	1.86 (1.29, 2.68)^f^	1.64 (1.12, 2.39)^d^	3.24 (2.25, 4.66)^f^	0.046
Dyslipidemia							
aOR^c^_[Yes vs. No]_, (95% CI)	1.68 (1.28, 2.20)^f^	2.01 (1.56, 2.57)^f^	2.45 (1.63, 3.68)^f^	2.74 (1.93, 3.90)^f^	1.25 (0.87, 1.80)	1.49 (1.04, 2.13)^d^	0.016
Total cholesterol							
aOR^c^_[Borderline vs. Acceptable]_, (95% CI)	1.41 (0.93, 2.13)	1.99 (1.37, 2.88)^f^	2.53 (1.09, 5.86)^d^	5.62 (2.80, 11.27)^f^	1.22 (0.75, 2.00)	1.01 (0.60, 1.70)	< 0.001
aOR^c^_[Abnormal vs. Acceptable]_, (95% CI)	0.79 (0.35, 1.80)	1.81 (0.99, 3.38)	3.54 (0.82, 15.23)	5.42 (1.48, 19.93)^d^	0.35 (0.10, 1.21)	1.14 (0.51, 2.54)	
Proportional^g^ aOR^c^, (95% CI)	1.22 (0.84, 1.79)	1.94 (1.39, 2.69)^f^	2.76 (1.33, 5.76)^e^	5.45 (2.92, 10.16)^f^	0.95 (0.60, 1.51)	1.06 (0.68, 1.66)	< 0.001
LDL cholesterol							
aOR^c^_[Borderline vs. Acceptable]_, (95% CI)	1.85 (0.81, 4.20)	4.05 (2.06, 7.95)^e^	2.53 (0.66, 9.69)	5.84 (1.93, 17.70)^e^	1.55 (0.54, 4.45)	3.11 (1.28, 7.58)^d^	0.357
aOR^c^_[Abnormal vs. Acceptable]_, (95% CI)	1.67 (0.57, 4.90)	3.40 (1.41, 8.16)^f^	5.45 (1.03, 29.97)^d^	6.60 (1.42, 30.74)^d^	0.39 (0.05, 3.27)	2.39 (0.74, 7.70)	
Proportional^g^ aOR^c^, (95% CI)	1.78 (0.92, 3.44)	3.79 (2.21, 6.51)^f^	3.51 (1.24, 9.95)^d^	6.11 (2.47, 15.16)^f^	1.07 (0.43, 2.70)	2.81 (1.37, 5.75)^e^	0.228
Non‐HDL cholesterol							
aOR^c^_[Borderline vs. Acceptable]_, (95% CI)	1.75 (1.08, 2.81)^d^	2.67 (1.76, 4.06)^f^	2.75 (1.24, 6.11)^d^	4.47 (2.26, 8.86)^f^	1.37 (0.75, 2.52)	1.82 (1.03, 3.23)^d^	0.131
aOR^c^_[Abnormal vs. Acceptable]_, (95% CI)	3.00 (1.24, 7.25)^d^	5.73 (2.65, 12.41)^f^	7.77 (1.57, 38.37)^d^	15.17 (3.45, 66.71)^f^	1.69 (0.52, 5.42)	2.92 (1.06, 8.07)^d^	
Proportional^g^ aOR^c^, (95% CI)	1.97 (1.29, 3.00)^e^	3.23 (2.23, 4.68)^f^	3.51 (1.73, 7.14)^f^	6.03 (3.25, 11.21)^f^	1.42 (0.82, 2.46)	2.04 (1.23, 3.38)^e^	0.025
HDL cholesterol							
aOR^c^_[Borderline vs. Acceptable]_, (95% CI)	1.54 (1.14, 2.09)^e^	1.77 (1.33, 2.36)^f^	1.39 (0.87, 2.22)	1.49 (0.98, 2.26)	1.61 (1.07, 2.42)^d^	2.07 (1.39, 3.07)^f^	0.575
aOR^c^_[Abnormal vs. Acceptable]_, (95% CI)	2.06 (1.39, 3.03)^f^	2.97 (2.10, 4.20)^f^	1.67 (1.01, 2.78)^d^	2.63 (1.70, 4.07)^f^	2.82 (1.50, 5.32)^e^	3.27 (1.75, 6.12)^f^	
Proportional^g^ aOR^c^, (95% CI)	1.65 (1.28, 2.14)^f^	2.17 (1.71, 2.75)^f^	1.46 (1.01, 2.10)^d^	2.08 (1.51, 2.85)^f^	1.87 (1.30, 2.68)^f^	2.25 (1.58, 3.20)^f^	0.633
Triglycerides							
aOR^c^_[Borderline vs. Acceptable]_, (95% CI)	1.74 (1.26, 2.41)^f^	1.97 (1.45, 2.68)^f^	1.50 (0.90, 2.48)	2.14 (1.39, 3.31)^f^	1.93 (1.27, 2.94)^e^	1.77 (1.15, 2.70)^e^	0.089
aOR^c^_[Abnormal vs. Acceptable]_, (95% CI)	1.92 (1.38, 2.68)^f^	2.32 (1.71, 3.17)^f^	2.58 (1.57, 4.23)^f^	3.20 (2.06, 4.97)^f^	1.51 (0.96, 2.37)	1.71 (1.10, 2.66)^d^	
Proportional^g^ aOR^c^, (95% CI)	1.63 (1.28, 2.08)^f^	1.86 (1.49, 2.33)^f^	2.09 (1.45, 3.02)^f^	2.35 (1.71, 3.23)^f^	1.37 (1.00, 1.89)^d^	1.49 (1.08, 2.05)^d^	0.080

Abbreviations: aOR, adjusted odds ratio; CI, confidence interval; HDL‐C, high‐density lipoprotein cholesterol; LDL‐C, low‐density lipoprotein cholesterol.

^a^ Under/normal weight body mass index‐for age category was the common reference group.

^b^ Assessing the interaction between sex and body mass index‐for‐age categorical variables in the total study sample.

^c^ In the total study sample: Adjusted for sex, age, nationality, breastfeeding status, smoking status, physical activity status, and parental history of diabetes. In the sex‐stratified analysis, adjusted for age, nationality, breastfeeding status, smoking status, physical activity status, and parental history of diabetes.

^d^ *p*‐value < 0.05.

^e^ *p*‐value < 0.01.

^f^ *p*‐value < 0.001.

^g^ Ordinal logistic regression was used to estimate proportional odds across ordered categories of the outcome variable (acceptable, borderline, and abnormal).

**FIGURE 1:**
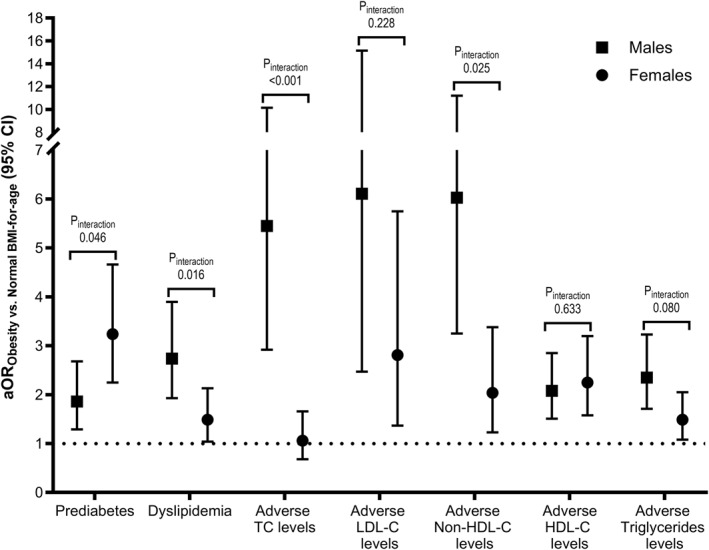
Sex‐stratified associations between obesity and prediabetes, dyslipidemia, and adverse lipid levels. Results of binary logistic regression models are presented for prediabetes and dyslipidemia as they are binary outcome variables. Results of ordinal logistic regression models (i.e., proportional odds ratios) are being presented for total cholesterol (TC), low‐density lipoprotein cholesterol (LDL‐C), high‐density lipoprotein cholesterol (HDL‐C), non‐HDL‐C, and triglycerides outcome variables. Under/normal weight body mass index (BMI)‐for age category was the common reference group. Odds ratios were adjusted for age, nationality, breastfeeding status, smoking status, physical activity status, and parental history of diabetes. *P*_interaction_ refers to the *p*‐value of the interaction term between the BMI‐for‐age categorical variable and sex in the total study sample (BMI‐for‐age × sex). aOR, adjusted odds ratio; CI, confidence interval.

In the total analytical sample, obesity compared with the underweight/normal BMI‐for‐age category was associated with higher odds of prediabetes (aOR: 2.53; 95% CI: 1.95, 3.27; Table 4). Nonetheless, the association between the BMI‐for‐age categories and prediabetes differed according to sex (*P*_interaction_ = 0.046). Overweight was associated with prediabetes among female participants (aOR: 1.64; 95% CI: 1.12, 2.39) but not among male participants. Obesity was associated with higher odds of having prediabetes among female subjects (aOR: 3.24; 95% CI: 2.25, 4.66) compared with male subjects (aOR: 1.86; 95% CI: 1.29, 2.68; Figure 1).

Although overweight and obesity were associated with increased odds of dyslipidemia in the total study sample, the association differed according to sex (*P*_interaction_ = 0.016). Overweight increased the odds of dyslipidemia among male participants (aOR: 2.45; 95% CI: 1.63, 3.68) but not among female participants (Table 4). Obesity in male subjects (aOR: 2.74; 95% CI: 1.93, 3.90) compared to obesity in female subjects (aOR: 1.49; 95% CI: 1.04, 2.13) was associated with higher odds of dyslipidemia in reference to underweight/normal weight (Figure 1). Similar sex‐dependent associations between the BMI‐for‐age categories and TC were observed (*P*_interaction_ < 0.001), with obesity compared to underweight/normal weight being associated with higher odds of having adverse TC levels in male participants (proportional aOR: 5.45; 95% CI: 2.92, 10.16), whilst overweight and obesity were not associated with TC in female participants (Figure 1).

Similarly, the association between obesity and abnormal LDL‐C levels was stronger among male subjects (proportional aOR: 6.11; 95% CI: 2.47, 15.16) than female subjects (proportional aOR: 2.81; 95% CI: 1.37, 5.75); nonetheless, this observed sex‐related difference was not statistically significant (*P*_interaction_ = 0.228; Figure 1). Moreover, associations of BMI‐for‐age categories with non‐HDL‐C (*P*_interaction_ = 0.025) and triglycerides (*P*_interaction_ = 0.080) differed between male and female participants. For instance, obesity compared with underweight/normal weight demonstrated a stronger association with abnormal levels of non‐HDL‐C in male participants (proportional aOR: 6.03; 95% CI: 3.25, 11.21) compared with female participants (proportional aOR: 2.04; 95% CI: 1.23, 3.38; Figure 1). In contrast, overweight and obesity were associated with abnormal HDL‐C levels in both male and female subjects to a similar magnitude (*P*_interaction_ = 0.633; Table 4).

## 4 Discussion

In this study, approximately one‐third of the adolescents were affected by prediabetes, and nearly half had dyslipidemia. Sex‐related differences in the association of obesity with prediabetes and dyslipidemia were also found. Specifically, obesity was a stronger risk factor for prediabetes among female participants than among male participants. Obesity was a stronger risk factor for dyslipidemia and abnormal lipid levels among male subjects than among female subjects. Such findings highlight the potential sex‐specific effects of obesity on metabolic conditions among adolescents.

The estimated prevalence of prediabetes (34.3%) indicates that a substantial proportion of the enrolled adolescents are affected and hence are susceptible to progression to T2DM and microvascular and macrovascular complications [30, 31]. A meta‐analysis among 6,630,296 subjects aged ≤ 20 years old from a total of 48 studies estimated the prevalence of prediabetes to be 8.84%, with prevalence estimates ranging between 0.25% and 23.47% [32]. Among US adolescents (aged 12–19 years), the prevalence of prediabetes was estimated to be 36.3% in 2015–2020 [33]. In this study, the prevalence of prediabetes did not differ according to sex, which is in line with the finding of a large meta‐analysis [32]. Nonetheless, other studies have shown contradicting findings regarding sex differences in prediabetes prevalence among adolescents [17, 33, 34, 35]. Factors that may contribute to the observed conflicting findings can be the characteristics of the study population (e.g., overweight/obesity levels, age, and race/ethnicity) and the used diagnostic test.

According to previous studies, the clinical manifestation of prediabetes differs between males and females. Males tend to display elevations in fasting plasma glucose more often, whereas females show higher postprandial glucose levels [36]. An increase in fasting glucose is due to the liver producing more glucose and a reduction in early insulin secretion, whereas postprandial hyperglycemia is primarily caused by peripheral insulin resistance [36]. Even though fasting glucose, postprandial glucose, and insulin resistance were not measured in this study, the insulin resistance, and the higher occurrence of postprandial plasma glucose reported in females in other studies and its stronger correlation with HbA1c [37] may explain why obesity was a stronger risk factor for prediabetes among female (aOR: 3.24) compared to male (aOR: 1.86) participants in this study. Supporting the findings of the current study, a previous study assessing the association between obesity during adolescence and incident T2DM in early adulthood showed higher obesity‐related risk estimates for T2DM in females compared to male participants [38]. Additionally, males were significantly more active than females in the current study (Table 1), and exercise has been shown to improve insulin sensitivity among overweight adolescents with prediabetes through multiple mechanisms such as enhanced glucose uptake, reduction in visceral fat, improved mitochondrial function, and decreased systemic inflammation [39].

Moreover, this study estimated that 45.1% of the enrolled adolescents were affected by dyslipidemia. The estimated prevalence of dyslipidemia in the current study is higher than a reported dyslipidemia prevalence estimate among Saudi adolescents of 25.5% [40]. Among US adolescents (aged 12–19 years), the prevalence of having at least one adverse level of HDL‐C, non‐HDL‐C, or TC was estimated to be 21.8% in 2013–2016 [41]. The prevalence of dyslipidemia among American Indian adolescents (aged 15–19 years) was estimated to be 55.2% [42], which is similar to the estimated dyslipidemia prevalence in the current report. The observation of frequent abnormal levels of HDL‐C (19.7%) and triglycerides (33.0%) is supported by findings from a systematic review by the US Preventive Services Task Force, which showed abnormal levels of HDL‐C (range: 12.1%–22.2%) and triglycerides (range: 8.0%–17.3%) being more common than other lipid parameters among children and adolescents [43]. A regional systematic review based on Iranian children and adolescents also reported that abnormal levels of HDL‐C (range: 5%–88%) and triglycerides (3%–50%) were the most prevalent lipid abnormalities in their study population [44]. Such abnormal lipid levels during early‐life have been shown to carry into adulthood and are associated with increased risk of cardiovascular diseases [45, 46].

Sex differences in the prevalence of dyslipidemia and abnormal lipid measures were identified in the current study, with more male (54.2%) than female (36.7%) participants being affected by dyslipidemia. This sex dimorphism in lipid levels has been reported by previous studies and attributed to sex chromosomes, sex hormones, sex‐biased genetic regulation, female reproductive stages, and lifestyle factors [40, 47].

Additionally, obesity showed stronger associations with dyslipidemia and abnormal lipid levels in males compared with female adolescents in this study. For instance, obesity showed a stronger association with dyslipidemia (aOR: 2.74 vs. 1.49) and abnormal levels of TC (proportional aOR: 5.45 vs. 1.06) and non‐HDL‐C (proportional aOR: 6.03 vs. 2.04) in males compared with females. These findings are in agreement with the study by Skinner et al., which showed that obesity is associated with a higher prevalence of lipid abnormalities in males compared with female adolescents [48]. The mechanism underlying the observed sex differences in the obesity‐dyslipidemia relationship could also be attributed to sex differences in the accumulation of VAT during puberty, which is more common in males than in females [49]. VAT compared with SAT poses a greater risk of cardiometabolic disease through increased secretion of pro‐inflammatory molecules causing a state of systemic, low‐grade, chronic inflammation [4, 15]. According to a study by Lemieux et al., adjustment for abdominal visceral fat area removed differences in triglyceride levels between adult males and females [50].

One of the study's strengths is its large, representative sample of adolescents in Kuwait. The measurement of lipid levels using non‐fasting capillary blood constitutes a limitation of the study that may have led to the misclassification of lipid status. Nonetheless, existing evidence in both children and adults suggests marginal differences between fasting and non‐fasting lipid profiles, with some variations reported in triglyceride levels, though these are deemed negligible [51, 52]. Hence, the results of the current analysis might be minimally affected by the non‐differential misclassification of lipid status. This non‐differential misclassification will most probably underestimate the magnitude of the estimated measures of associations. With regard to the assessment of prediabetes, the current study only looked at HbA1c. The absence of data on oral glucose tolerance testing, fasting plasma glucose, postprandial plasma glucose, or insulin resistance represents a limitation. The lack of information on dietary intake is a further limitation in the current analysis. Moreover, lack of information on body composition measures (e.g., bioelectrical impedance) that distinguish between fat and lean mass is a further limitation, which would have provided a more detailed characterization of the observed sex‐specific effects on prediabetes and dyslipidemia. Causality should not be inferred from the reported results because the study assessed concurrent associations, and thus temporality is not established.

## 5 Conclusion

The elevated prevalence of prediabetes and dyslipidemia and the increased odds of these conditions with obesity among adolescents pose a tremendous burden on public health and should be urgently addressed. In the current report, obesity demonstrated sex‐specific associations with prediabetes, dyslipidemia, and lipid measures. Specifically, stronger associations between obesity and prediabetes were observed among female participants compared with male participants, while associations between obesity and dyslipidemia and lipid measures were stronger among male participants. This highlights the fact that obesity may pose differential deleterious cardiometabolic effects in male and female adolescents. Therefore, future epidemiological studies are needed to corroborate and investigate the underlying biological and behavioral mechanisms driving these sex differences. Moreover, these results call for sex‐specific risk stratification strategies to effectively address the differential risks and offer greater potential for targeted prevention among adolescents.

## Author Contributions

A.H.Z. was responsible for conceiving, designing, and planning the study, as well as analyzing and interpreting the data, and drafted the initial manuscript. A.S. contributed to the data analysis and interpretation and helped draft the manuscript. Z.A. assisted with data interpretation and contributed to drafting and revising the manuscript. M.A. was instrumental in conceiving, designing, and planning the study, obtaining funding and supervising the fieldwork. T.A. contributed to data interpretation and manuscript revisions. All authors reviewed and approved the final manuscript and provided critical revisions for important intellectual content.

## Funding

This project was partially funded by the Kuwait National Guard, the College of Graduate Studies at Kuwait University, and Salman Abdullah Al Dabbous and Sons Company.

## Conflicts of Interest

The authors declare no conflicts of interest.

## Data Availability

The data that support the findings of this study are available on request from the corresponding author. The data are not publicly available due to privacy or ethical restrictions.
